# Mitochondrial Changes in β^0^-Thalassemia/Hb E Disease

**DOI:** 10.1371/journal.pone.0153831

**Published:** 2016-04-19

**Authors:** Kornpat Khungwanmaythawee, Wannapa Sornjai, Atchara Paemanee, Janejira Jaratsittisin, Suthat Fucharoen, Saovaros Svasti, Pathrapol Lithanatudom, Sittiruk Roytrakul, Duncan R. Smith

**Affiliations:** 1 Institute of Molecular Biosciences, Mahidol University, Salaya campus, 25/25 Phuttamontol Sai 4, Salaya, Nakorn Pathom, 73170, Thailand; 2 National Center for Genetic Engineering and Biotechnology (BIOTEC), National Science and Technology Development Agency, 113 Thailand Science Park, Phahonyothin Road, Khlong Nueng, Khlong Luang, Pathum Thani, 12120, Thailand; 3 Department of Biology, Faculty of Science, Chiang Mai University, 239 Huay Kaew Rd., Suthep, Muang, Chiang Mai, 50202, Thailand; University of Palermo, ITALY

## Abstract

The compound β°-thalassemia/Hb E hemoglobinopathy is characterized by an unusually large range of presentation from essentially asymptomatic to a severe transfusion dependent state. While a number of factors are known that moderate presentation, these factors do not account for the full spectrum of presentation. Mitochondria are subcellular organelles that are pivotal in a number of cellular processes including oxidative phosphorylation and apoptosis. A mitochondrial protein enriched proteome was determined and validated from erythroblasts from normal controls and β°-thalassemia/Hb E patients of different severities. Mitochondria were evaluated through the use of mitotracker staining, analysis of relative mitochondrial genome number and evaluation of mitochondrial gene expression in addition to assay of overall cellular redox status through the use of alamarBlue assays. Fifty differentially regulated mitochondrial proteins were identified. Mitotracker staining revealed significant differences in staining between normal control erythroblasts and those from β°-thalassemia/Hb E patients. Differences in relative mitochondria number and gene expression were seen primarily in day 10 cells. Significant differences were seen in redox status as evaluated by alamarBlue staining in newly isolated CD34+ cells. Mitochondria mediate oxidative phosphorylation and apoptosis, both of which are known to be dysregulated in differentiating erythrocytes from β°-thalassemia/Hb E patients. The evidence presented here suggest that there are inherent differences in these cells as early as the erythroid progenitor cell stage, and that maximum deficit is seen coincident with high levels of globin gene expression.

## Introduction

The thalassemias are a diverse group of hematological disorders arising from the inheritance of defective globin genes [1]. The compound β°-thalassemia/Hb E hemoglobinopathy is common in Southeast Asia, and is characterized by a wide range of presentation from essentially asymptomatic to a severe, transfusion dependent condition [2]. While a number of factors including the co-inheritance of α-globin hemoglobinopathies and the level of HbF, have been shown to play a role in modulating the presentation of the disease, these factors do not account completely for the range of presentation found for apparently comparable underlying genetic lesions [2], suggesting that other factors remain to be found that modulate the presentation of the disease.

The primary characteristic of β°-thalassemia/ Hb E is anemia of a variable severity. The anemia arises from a combination of ineffective erythropoeisis and increased hemolysis of the mature red blood cells [3, 4]. Ineffective erythropoiesis occurs as a consequence of apoptosis occurring at the polychromatophilic normoblast stage of erythropoiesis [3], and the small proportion of cells that do mature to red blood cells undergo increased hemolysis as a consequence of the deposition of unpaired α-globin chains in the cells [4]. As a consequence of the anemia, levels of erythropoietin are increased [5] leading to expansion of the erythroid mass, but because of ineffective erythropoiesis this does not result in significant alleviation of the anemic state. Previous studies have noted that cultured erythroid progenitor cells from β°-thalassemia/Hb E patients show increased cell expansion and increased differentiation as compared to erythroid progenitor cells from normal controls [6]. The reasons for the increased expansion of β°-thalassemia/Hb E erythroid precursor cells remain unclear. It is possible that this results from some form of conditioning in which the progenitor cells from thalassemia patients are primed to undergo increased expansion as a consequence of the higher levels of EPO in the patients from which the cells are taken, or alternatively the increased expansion could reflect an inherent difference in the cells.

In a previous study we undertook a proteomic analysis of erythroid precursor cells from both normal controls and β°-thalassemia/Hb E patients [7]. That study showed increased levels of a number of proteins in β°-thalassemia/ Hb E erythroid precursor cells, of which the largest single class was proteins involved in glycolysis and the tricarboxylic acid (TCA) cycle. We additionally demonstrated increased levels of oxidative phosphorylation in these cells [7]. The TCA cycle and oxidative phosphorylation occur in the mitochondria, a maternally inherited subcellular organelle [8]. Studies have shown that mitochondria vary greatly in their numbers and in their activity depending upon the energy requirements of the cell [9]. Mitochondria have their own genetic material which encodes for some 37 genes of which code for 2 ribosomal RNAs (rRNAs), 22 for transfer RNAs (tRNAs) and 13 for polypeptides which are core protein subunits of the oxidative phosphorylation system (comprehensively reviewed by Friedman and Nunnari [10]). The majority of proteins involved with the TCA cycle and oxidative phosphorylation are therefore genes encoded in the cellular genome, and not the mitochondrial genome, and these proteins determine metabolic function and activity of the mitochondria [11]. Given the association with the TCA cycle and oxidative phosphorylation, processes that we have shown disordered in β-thalassemia/ Hb E erythroid cells [7], this study sought to take a more detailed look at mitochondria and their relationship with β-thalassemia/Hb E disease.

## Materials and Methods

### Patients, Sample Collection and Erythroid Cell Culture

This study was approved by the Ethical committee, Mahidol University Institutional Review Board (IRB 2009/038.0202). Written informed consent was obtained from participants before sample collection. Patients were identified, disease severity graded and controls were screened as previously [6]. Fifty ml of peripheral blood was taken from healthy controls and 25 ml of peripheral blood was taken from patients. CD34+ cells were isolated from peripheral blood and cultured in supplemented Iscove’s modified Dulbecco medium as previously described [6]. Cell numbers were determined by trypan blue staining using a hemocytometer.

### Protein Preparation and GeLC-MS/MS

Mitochondria were purified from day 10 erythrocytes from 5 normal controls, 5 mild and 5 severe β°-thalassemia/Hb E patients using magnetically labeled anti-TOM22 microbeads (Miltenyi Biotech, Auburn, CA) and separation on an LS Column placed in a MidiMACS Separator (Miltenyi Biotech) according to the manufacturers protocol. Mitochondria preparations were lysed with 0.5% SDS and sonicated for 5 min twice. Proteins from each group were pooled and subsequently analyzed in duplicate. GeLC-MS/MS and protein identification was undertaken exactly as previously described [12], except that the Mitoproteome database [13] was searched in addition to the NCBI database.

### Western Blotting

Western blotting was undertaken essentially as described elsewhere [7]. A total of 30μg of proteins were run per lane on 12% SDS-PAGE gels. Primary antibodies used included a 1:1000 dilution of a rabbit polyclonal anti-Prohibitin 2 (Santa Cruz Biotechnology, Santa Cruz, CA) antibody and a 1:1000 dilution of a rabbit polyclonal anti-HSP60 antibody (Santa Cruz Biotechnology).

### Mitotracker Staining

Erythroid precursor cells were incubated with 500 nM MitoTracker Red CMX Ros (MTR, Molecular Probes, Eugene, OR) for 15 min following which cells washed with PBS-IFA (0.15M NaCl, 0.05M NaH_2_PO_4_, 0.05M Na_2_HPO_4_, pH 7.4), cytocentrifuged onto glass slides, fixed with 4% paraformaldehyde, permeablilized with 0.3% tritonX/PBS and counterstained with DAPI (Merck Millipore, Temecula, CA) before visualization under a confocal microscope. For flow cytometry cells were stained with mitotracker as above, washed with PBS-IFA and analyzed directly by flow cytometry (BD, FACSCalibur) using the CELLQuest software (BD Biosciences).

### AlamarBlue Assay

AlamarBlue (Thermo Fisher Scientific, Waltham, MA) assays were undertaken according to the manufacturers’ protocol on 1 x 10^4^ cells.

### Quantitative Real-Time PCR

At least 1x10^6^ cells were collected by centrifugation, washed with 1x ice-cold PBS and re-centrifuged. The cell pellet was resuspend in 1 ml of TRI reagent (Molecular Research Center, Inc., Cincinnati, OH) and DNA and RNA prepared according to the manufacturer’s instructions. mRNA was reverse transcribed using random primers and ImpromII^™^ reverse transcriptase (Promega, Madison, WI). Real-time PCR amplification (first strand cDNA and DNA) was performed in 20μL containing 2X Kapa SYBR^®^ FastMaster Mix (Kapa Biosystems, Inc., Wilmington, MA), 300 nM each primer (Table A in S1 File) and 5ng (cDNA) or 50ng (DNA) template. Primers and cycle conditions are given in Table A in S1 File. mRNA expression data was normalized to β-actin, while mitochondrial DNA copy number was normalized to ferroportin 1A.

### Statistical Analysis

Statistical analysis was performed using the PASW statistics 18 (SPSS Inc. Chicago, IL). And all data was compared using independent sample *t*-tests. Data was considered as a statistical significant at a *p* value of less than 0.05.

## Results

### Mitochondria Enriched Proteome Analysis

**Fig 1:**
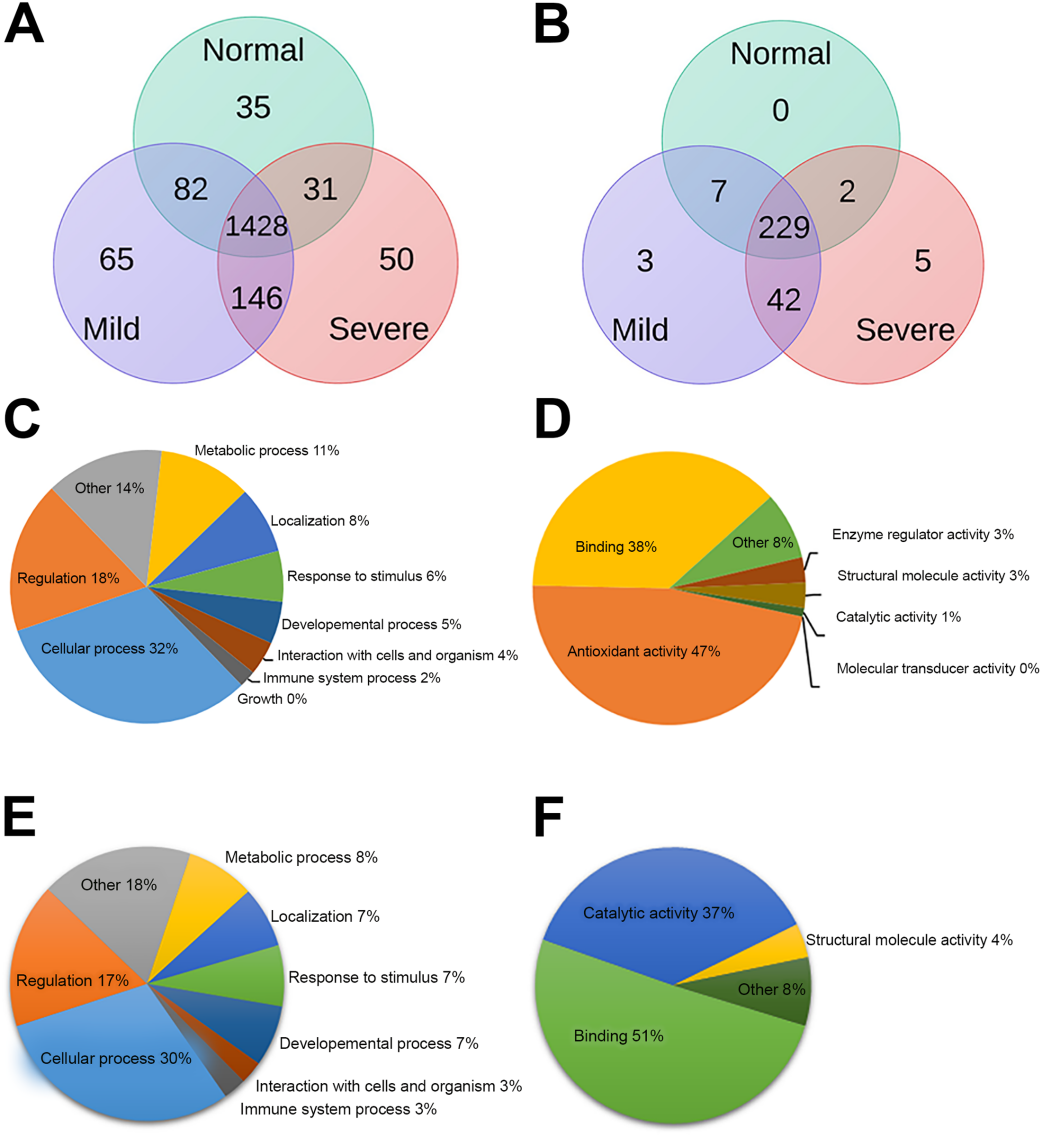
The mitochondrial protein enriched proteome of erythroid cells. Mitochondrial protein enriched preparations from day 10 erythroid cells from normal controls and β°-thalassemia/Hb E patients (mild and severe) were subjected to GelC-MS/MS analysis. Venn diagrams of (a) total proteins identified and (b) mitochondrial proteins as identified by the Mitoproteome database and GoCat ontological analysis of (c) cellular processes and (d) functional categorization of the identified mitochondrial proteins and GoCat ontological analysis of (e) cellular processes and (f) functional categorization of the identified differentially regulated proteins.

**Fig 2:**
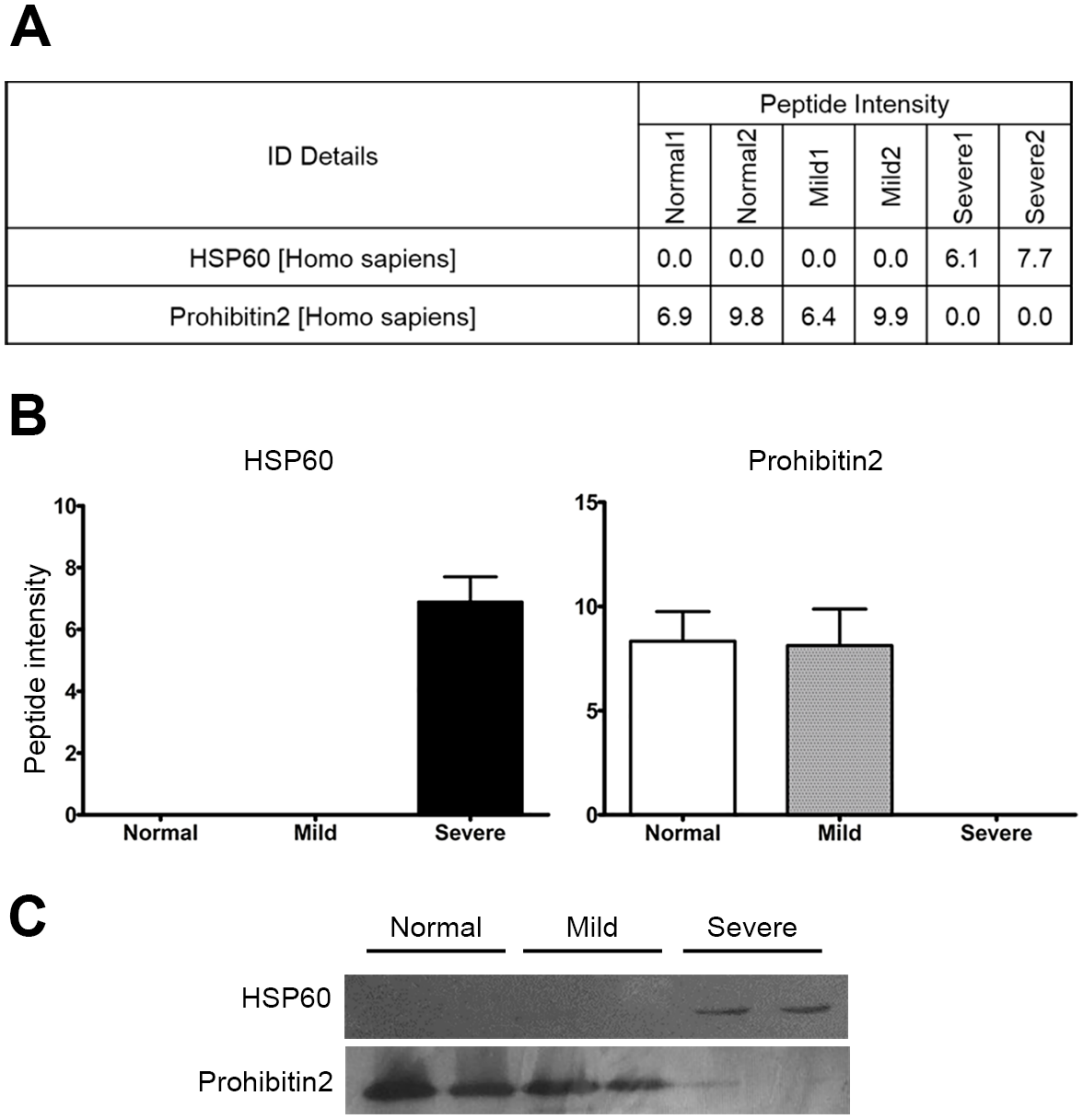
Validation of proteome data. Based on the proteome data, two differentially regulated mitochondrial proteins (hsp60 and prohibitin 2) were selected for validation in an independent cohort of controls and patients. Raw proteome data reads (a) and quantitation (b) are shown together with (c) western blot analysis of the independent cohort. Samples were pooled from 5 individuals and run as duplicate lanes.

To investigate the profiles of mitochondria associated proteins in β°-thalassemia/Hb E patients as compared to normal controls, mitochondria enriched proteins were obtained from day 10 erythroblasts of five normal controls, five severe and five mild β°-thalassaemia/Hb E patients, using anti-TOM22 microbeads to purify mitochondria followed by protein extraction. The protocol was shown to significantly enrich for mitochondrial proteins as shown by Western blotting for NADH:ubiquinone oxidoreductase subunit A9 (NDUFA9; Fig A in S1 File). The proteins were analyzed by a gel-enhanced liquid chromatography tandem mass spectroscopy (GeLC-MS/MS) [14]. Original gels are shown in Fig B in S1 File. Resultant analysis of the generated spectra identified 4392 peptides corresponding to 1837 proteins, of which some 1428 were not differentially expressed between samples (Fig 1a). Ontological analysis of these proteins for biological process and molecular function is shown in Fig 1c and 1d. The spectra were subsequently screened against the Mitoproteome database on 10^th^ and 19^th^ June 2013 which identified some 288 mitochondrial proteins, representing approximately 40% of the database annotated mitochondrial proteome. Of these 288 proteins, three proteins showed significantly increased expression in mild β°-thalassemia/Hb E patients only, while a further five proteins showed significantly increased expression in severe β°-thalassemia/Hb E patients only. A total of forty-two proteins were significantly up-regulated in both mild and severe β°-thalassemia/Hb E patients as compared to normal controls (Fig 1b and Table B in S1 File). The heat map is shown in Fig C in S1 File. Interestingly, no protein was detected as showing down-regulation in β°-thalassemia/Hb E erythroblasts as compared to normal control erythroblasts. To validate the proteomic data, western blot analysis of two proteins identified as differentially expressed was undertaken on samples from an independent cohort of patients and controls. The proteins selected, heat shock protein 60 (hsp60) and prohibitin2 are both well characterized mitochondrial chaperone proteins and both have been shown to have additional, albeit predominantly antagonistic, roles in the regulation of apoptosis (reviewed in [15]). As the proteome data showed discordant expression of these two proteins (hsp60 up in severe and prohibitin 2 down in severe) despite their similar chaperone functions their expression profile was considered to be important to validate. Both proteins showed results consistent with the proteome data (Fig 2).

The 50 proteins identified as being significantly up-regulated in β°-thalassemia/Hb E patients belonged to a number of cellular processed including oxidative phosphorylation and energy metabolism (Table B in S1 File). Ontological analysis using the GoCat software [16] showed that most of the differentially expressed proteins were involved in cellular processes (30%), regulation (17%) and metabolic processes (8%) as shown in Fig 1e. Functional categorization of the significantly differentially expressed proteins indicated up to 51% of the proteins were characterized as binding while 37% were characterized as having catalytic activity (Fig 1f**)**.

### Mitochondria in β°-Thalassemia/Hb E

**Fig 3:**
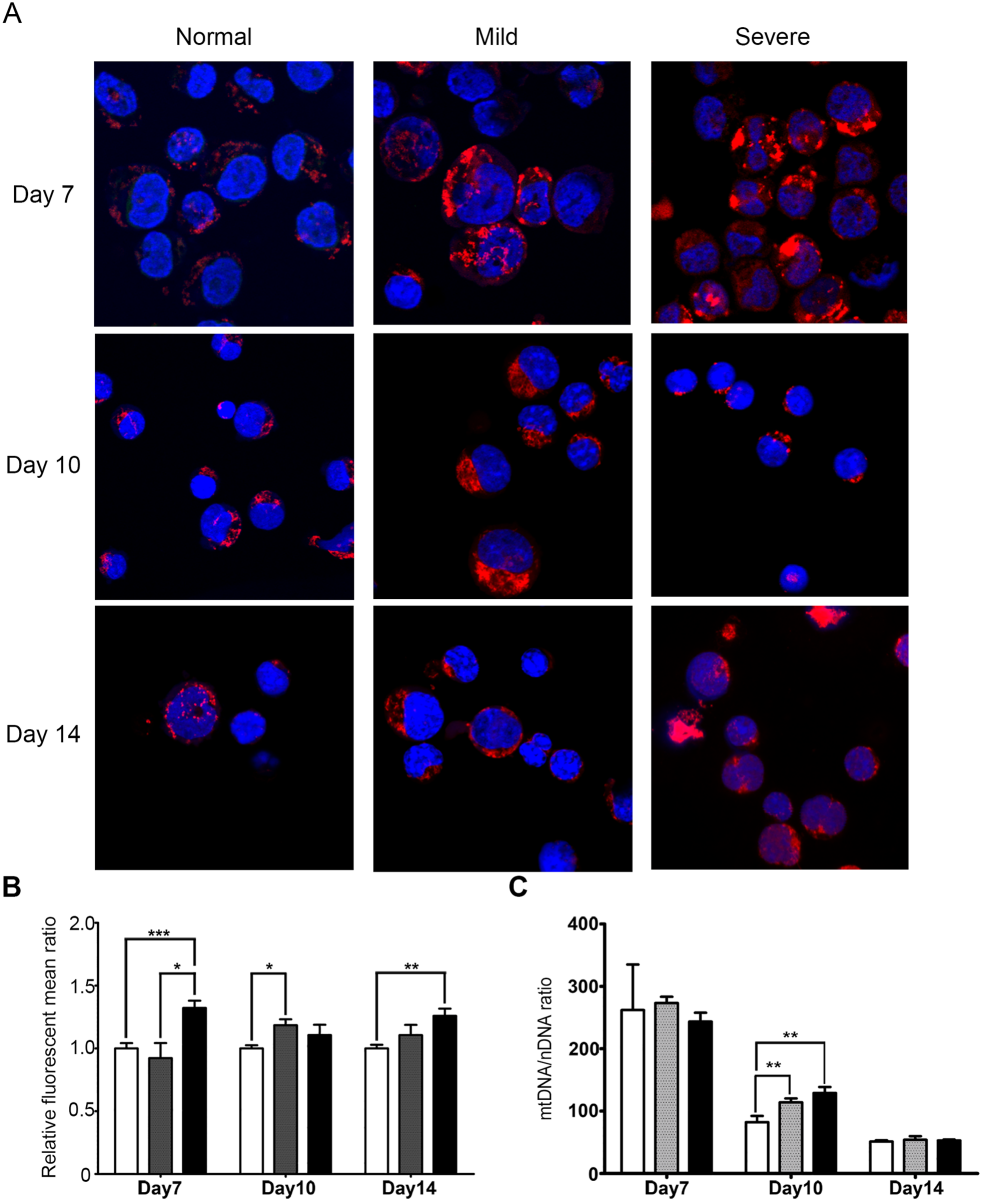
Mitochondria in erythroid precursor cells: quantity. Erythroid precursor cells on days 7, 10 and 14 of culture were (a and b) stained with mitotracker and examined by (a) confocal microscopy or (b) flow cytometry. (a) Representative merged confocal images are shown, and individual fields and merges are shown in Fig D in S1 File. (b) Tabulated flow cytometry data is shown, and individual scatterplots are shown in Fig E in S1 File. (c) Real-time quantitative PCR was used to determine relative mitochondria numbers. Data is normalized to nuclear genome through the ferroportin 1A gene. (b and c) white bars represent normal controls, grey bars are from mild and black bars are from severe β°-thalassemia/Hb E patients. Error bars show ± S.E.M. * p ≤ 0.05, ** p ≤ 0.01, ***p ≤ 0.001.

Mature red blood cells do not contain mitochondria [17] and it is well established that mitochondria are lost by the process termed mitophagy (reviewed by Mortensen and colleagues [18]), and it is generally believed that mitochondria are lost during reticulocyte maturation [17]. To directly observe mitochondria during erythroblast differentiation cells on days 7, 10 and 14 were stained with mitotracker and observed under a confocal microscope. Results showed markedly different patterns of staining between cells from normal controls and those from β°-thalassemia/Hb E patients (Fig 3). This was most clearly observed on day 7 of culture whereby the signal in normal control cells was predominantly perinuclear, while in cells from β°-thalassemia/Hb E patients the signal was largely colocalized with the nuclei (Fig 3).

Quantitative analysis of the signal by flow cytometry showed significant differences in the levels of signal between normal control cells and those from β°-thalassemia/Hb E patients (Fig 3). Mitotracker dyes are cationic fluorophores that accumulate in mitochondria as a consequence of the negative mitochondrial membrane potential, and inside the mitochondria the dye forms covalent bonds with proteins and peptides [19]. However, studies have shown that mitotracker dyes preferentially label selected proteins, and while the full list of preferential proteins is not know, studies have shown that hsp60 is a major labeling target of mitotracker dyes [20]. As shown in both the proteome analysis and in the western blotting validation with an independent sample cohort, hsp60 is highly differentially regulated between normal control erythroblasts and erythroblasts from β°-thalassemia/Hb E patients. Thus, results on quantitation of mitochondria using mitotracker need to be interpreted with extreme caution.

To provide an alternative method of quantifying levels of mitochondria, the relative levels of mitochondrial genomes were determined using quantitative PCR, with normalization against the nuclear genome. Results (Fig 3) show equal relative numbers of mitochondria on days 7 and 14, but higher relative levels of mitochondria in cells from β°-thalassemia/Hb E patients (both mild and severe) on day 10 of culture. Overall, the trend for all samples was consistent with the gradual loss of mitochondria during differentiation, rather than a loss of these organelles only during reticulocyte maturation.

**Fig 4:**
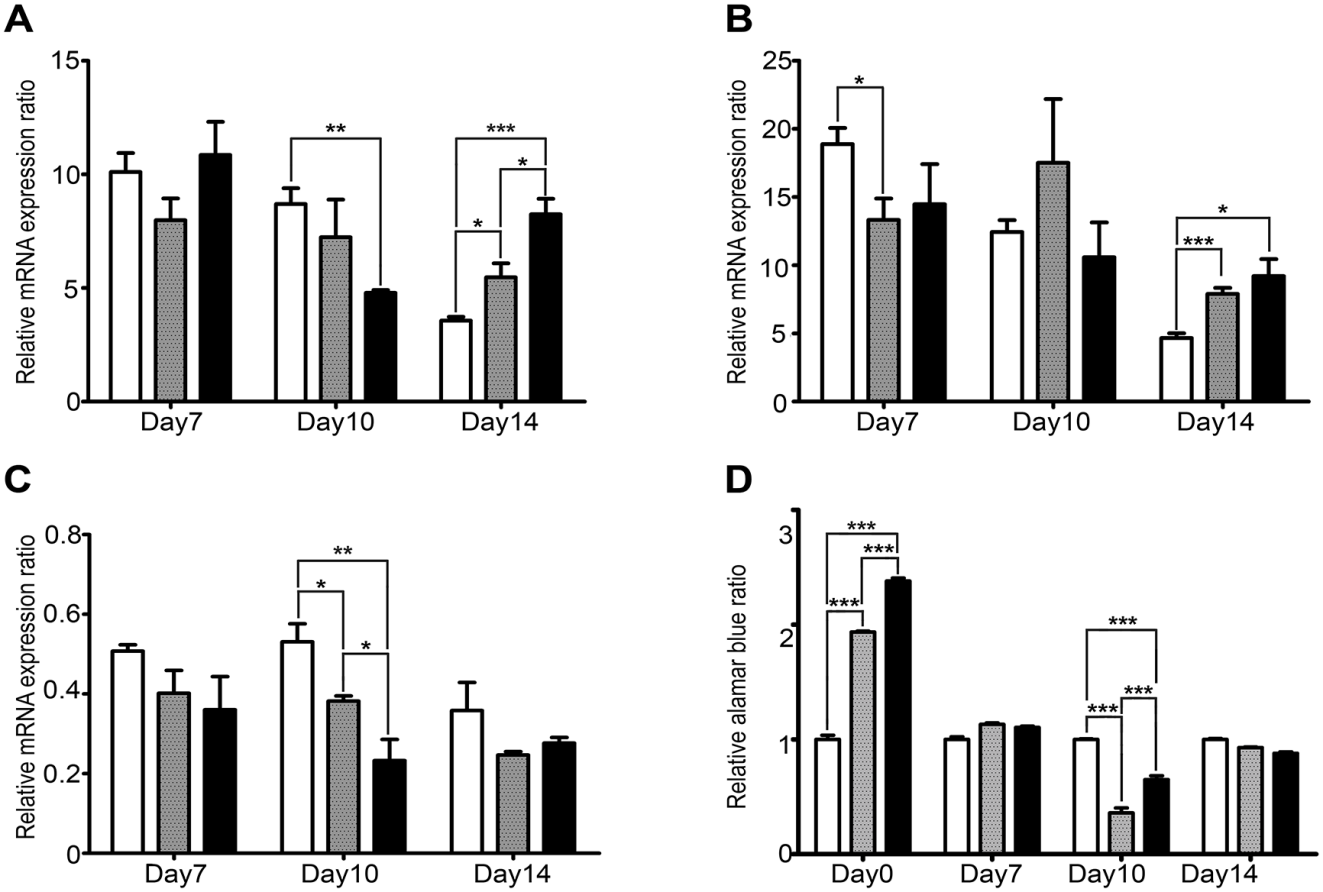
Mitochondria in erythroid precursor cells: activity. Day 7, 10 and 14 erythroid precursor cells from normal controls (white bars) and from mild (grey bars) and severe (black bars) β°-thalassemia/Hb E patients were examined for expression of (a) ATP6, (b) ATP8 or (c) CYTB by real time PCR on days 7, 10 and 14 of culture. (d) A total of 1 x10^4^ erythroid precursor cells from normal controls (white bars) and from mild (grey bars) and severe (black bars) β°-thalassemia/Hb E patients were used in alamarBlue assays. Error bars show ± S.E.M. * p ≤ 0.05, ** p ≤ 0.01, ***p ≤ 0.001.

Studies have suggested that mitochondria can be heterogeneous with respect to their activity. We therefore used quantitative PCR to determine relative expression levels of three mitochondrially encoded genes, namely ATP synthase F0 subunits 6 and 8 (ATP6 and ATP8) and mitochondrial cytochrome b (CYTB). Results (Fig 4) showed significant differences during differentiation for all three genes investigated. In particular expression of ATP6 and CTY were reduced in erythroblasts from severe β°-thalassemia patients as compared to normal controls on day 10 of culture. Interestingly both ATP6 and ATP8 were increased in expression in both mild and severe β°-thalassemia as compared to normal controls on day 14 of culture.

Resazurin (7-Hydroxy-3H-phenoxazin-3-one 10-oxide) is a commonly used oxidation reduction dye used to determine cell viability available commercially under a number of names including AlamarBlue. The assay measures the conversion of resazurin to resorufin which is mediated by mitochondrial reductases and other enzymes including NAD(P)H: quinine oxidoreductase, and reduction of AlamarBlue may signify impaired cellular metabolism, albeit not mitochondria specific dysfunction [21]. However, the AlamarBlue assay may be interpreted to give a read out of cellular redox status [21]. Constant numbers of erythroid progenitor (day 0 of culture) and erythroid precursor cells (days 7, 10 and 14 of culture) from normal controls and from β°-thalassemia/Hb E patients were therefore assayed by the AlamarBlue assay. Results (Fig 4) clearly show dysregulation in cells from thalassemia patients. In particular, significantly lower levels are seen on day 10 in cells from thalassemia patients. Interestingly however, cells on day 0 of culture (newly isolated CD34+ cells) show significantly higher levels (for both mild and severe patients) as compared to normal controls (Fig 4).

## Discussion

The size of the mitochondrial proteome is still poorly defined. The manually curated MitoProteome database lists 780 proteins as “current”, with a further 175 classified as “MS doubtful” and a further 317 as “doubtful”. However, it is clear that the mitochondrial proteome is highly variable by tissue [22], and some authors have proposed that there may be as many as 1500 mitochondrial proteins [23]. In our analysis mitochondria were purified through a magnetic bead isolation system, and therefore the samples analyzed represent a mitochondrially enriched proteome rather than a bone fide mitochondrial proteome. However, a total of 288 mitochondrial proteins were identified, of which some 50 showed differential regulation in β°-thalassemia/Hb E erythroblasts as compared to normal control erythroblasts. The proteome data was generated for day 10 of culture cells, representing a time point of significant globin expression as observed in our earlier proteomic analysis [7]. The proteins identified as differentially regulated included those associated with oxidative phosphorylation and energy metabolism, consistent with our earlier observations of dysregulation of these processes in β°-thalassemia/Hb E [7].

Two of the proteins identified as differentially expressed between normal controls and mild and severe β°-thalassemia/Hb E patients were hsp60 and prohibitin 2. Both of these proteins are well defined as mitochondrial proteins, although they also show localization in other cellular compartments, and both proteins are classed as mitochondrial chaperone proteins (reviewed in [15]). Somewhat surprisingly, the proteome data showed discordant expression of these two proteins, with hs60 showing increased expression in severe cases, while prohibitin2 was strongly down regulated in severe cases as compared to both normal controls and mild cases. Studies have shown that both of these proteins are involved in the regulation of apoptosis. Over expression of prohibitin increases cellular tolerance to stimuli that activate mitochondrially mediated apoptosis, while knock down of prohibitin broadly increases sensitivity to apoptosis, although cell type differences have been observed (reviewed in [24]). Hsp60 is believed to function as a pro-apoptotic molecule through its association with caspase-3 [25], although in cardiac myocytes the protein may negatively regulate apoptosis [26]. The main pathophysiology of β°-thalassemia/Hb E is mediated by ineffective erythropoeisis, which is the induction of apoptosis during erythroid differentiation at the polychromatophilic normoblast stage of erythroid differentiation [3]. In this regards the increased expression of the pro-apoptotic protein (hsp60) and the decreased expression of the anti-apoptotic protein (prohibitin2) seen in severe cases of β°-thalassemia/Hb E at day 10 of differentiation would appear to correlate with the main physiopathological process in severe cases of β°-thalassemia/Hb E.

Both confocal microscopy and flow cytometry showed higher levels of mitotracker staining from β°-thalassemia/Hb E erythroblasts as compared to normal control erythroblasts through the period of differentiation examined. However, as shown by confocal microscopy, the pattern of staining was discordant between controls and patients. In particular while day 7 erythroblasts showed essentially perinuclear staining, this was not observed in the thalassemic cells. However, studies have shown that mitotracker has a number of protein targets of which the most significant is hsp60 [20], a protein shown to be differentially regulated in this study, suggesting that quantitation of mitochondria in β°-thalassemia/Hb E should not be undertaken with mitotracker dyes.

Mitochondria are known to be removed during erythroid differentiation by the process of mitophagy and this is believed to occur during reticulocyte maturation [17]. However, our results with quantitation of relative genome copy number during differentiation show a consistent loss of mitochondria over the entire differentiation period. Interestingly, on day ten of differentiation, the relative numbers of remaining mitochondria in the thalassemic cells were higher than in normal controls, suggesting that mitochondria removal is slightly slower in these cells than in normal controls. This would again possibly be consistent with the increased oxidative phosphorylation see in these cells [7]. Interestingly, three autophagy/mitophagy related proteins (autophagy-related protein 13, E3 ubiquitin-protein ligase RNF 185 and ubiquitin-like modifier-activating enzyme ATG7) were seen as differentially expressed in the mitochondrial proteome, and as autophagy is up-regulated in β°-thalassemia/Hb E erythroblasts as compared to normal control erythroblasts [27], it suggests that selective mitophagy [28], rather than autophagy *per se* is disrupted.

While the relative numbers of mitochondria were higher in day 10 erythroblasts from β°-thalassemia/Hb E patients as compared to normal controls, in terms of activity a different picture emerged. In particular expression of two of the three mitochondrial genes examined was significantly lower on day 10 in the thalassemic cells. Consistent with this, an assessment cellular redox state through the alamarBlue assay showed a significantly decreased signal on day ten in the cells from β°-thalassemia/Hb E patients as compared to normal controls. Interestingly however, examination of newly isolated CD34+ erythroid progenitor cells by the alamarBlue assay showed a markedly higher redox status in both mild and severe β°-thalassemia cells as compared to normal controls, and markedly a significant difference between cells from mild and severe patients.

## Conclusion

Combined these results suggest a complex association between mitochondria and the pathology of β°-thalassemia/Hb E as mediated by erythroid differentiation. The results suggest that there is a markedly different redox state in newly isolated CD34+ cells, and this may result from the differing levels of EPO in these patients as compared to normal controls. Effects on mitochondria as seen by mitotracker staining are seen by day 7 of differentiation, and significant deficits in activity are seen on day 10, coincident with significant levels of globin chain synthesis [7]. These results would suggest that the deposition of unpaired globin chains is directly affecting the integrity of mitochondria. As there are more mitochondria present in cells from thalassemia patients on day 10, it suggests that the effect is magnified, with the damage to mitochondria at this point in time being co-incident with the onset of apoptosis in ineffective erythropoiesis [3].

## Supporting Information

S1 File Fig A. Western blot of NDUFA9 showing enrichment of mitochondrial proteins. Fig B. SDS-PAGE of mitochondria enriched proteins. Fig C. Hierarchical clustering analysis of 288 mitochondial proteins. Fig D. Original unmerged and merged images from Fig 3. Fig E. Original scatterplots of flow cytometry as presented in Fig 3. Table A. Specific primer sequences and cycle conditions. Table B. Significantly differentially expressed mitochondrial proteins in β°-thalassemia/Hb E erythroblasts.
(PDF)
