# Supplementary material for: Mitochondrial Changes in β0-Thalassemia/Hb E Disease
Source: PLoS One. 2016 Apr 19;11(4):e0153831. doi: 10.1371/journal.pone.0153831 (PMC4836671; doi:10.1371/journal.pone.0153831)
Supplement: S1 File — Fig A. Western blot of NDUFA9 showing enrichment of mitochondrial proteins. Fig B. SDS-PAGE of mitochondria enriched proteins. Fig C. Hierarchical clustering analysis of 288 mitochondial proteins. Fig D. Original unmerged and merged images from Fig 3. Fig E. Original scatterplots of flow cytometry as presented in Fig 3. Table A. Specific primer sequences and cycle conditions. Table B. Significantly differentially expressed mitochondrial proteins in β°-thalassemia/Hb E erythroblasts. (PDF) [file pone.0153831.s001.pdf]

S1 File

Mitochondrial Changes in β^0-Thalassemia/Hb E Disease

Kornpat Khungwanmaythawee[1], Wannapa Sornjai[1], Atchara Paemanee[1,2], Janejira Jaratsittisin[1], Suthat Fucharoen[1], Saovaros Svasti[1], Pathrapol Lithanatudom[3], Sittiruk Roytrakul[2] and Duncan R. Smith[1]*

[1]Institute of Molecular Biosciences, Mahidol University, Salaya campus, 25/25 Phuttamonton Sai 4, Salaya, Nakorn Pathom, Thailand 73170

[2]National Center for Genetic Engineering and Biotechnology (BIOTEC), National Science and Technology Development Agency, 113 Thailand Science Park, Phahonyothin Road, Khlong Nueng, Khlong Luang, Pathum Thani, Thailand 12120

[3]Department of Biology, Faculty of Science, Chiang Mai University, 239 Huay Kaew Rd., Suthep, Muang, Chiang Mai, Thailand 50202

*Corresponding author
Email: duncan.smi@mahidol.ac.th, duncan_r_smith@hotmail.com (DRS)

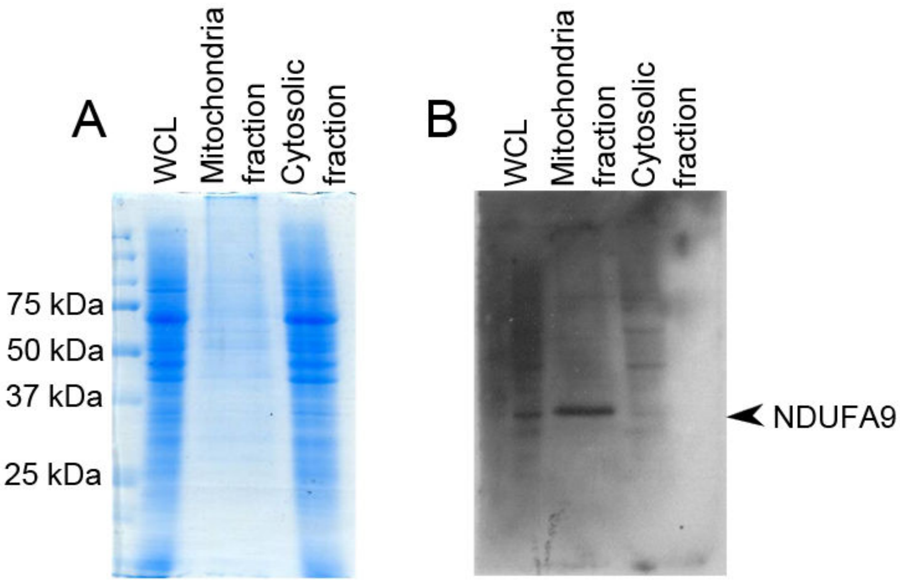

Fig A. Western blot analysis of mitochondrial protein enrichment.

Proteins were extracted from whole cell lysate (WCL), purified mitochondria and the cytosolic fraction obtained during the mitochondrial purification, and after electrophoresis through a 12.5% polyacrylamide gel were analyzed by (A) Coomassie blue staining and (B) Western blot analsyis to detect the presence of NADH:ubiquinone oxidoreductase subunit A9 (NDUFA9). Representative size markers are indicated.

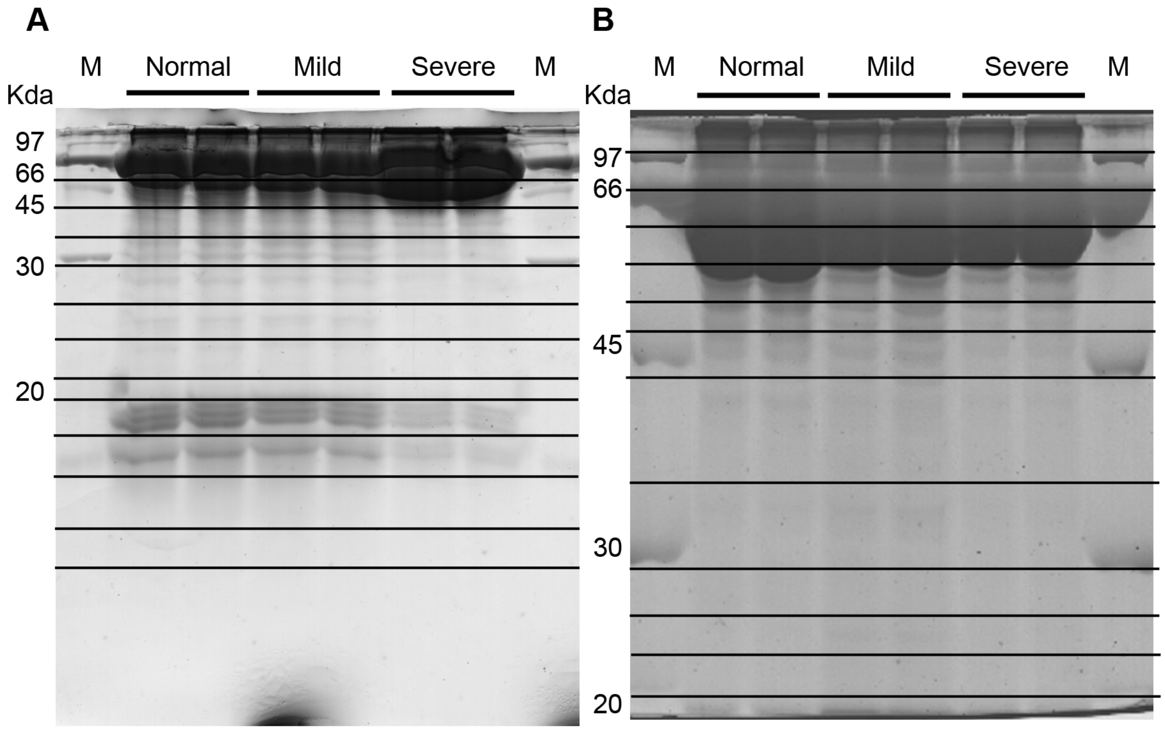

Fig B. SDS-PAGE of mitochondria enriched proteins.

Pools of mitochondrial protein enriched samples were prepared from 5 normal controls, 5 mild and 5 severe b-thalassemia/Hb E patients. A total of 50 μg from each pool was separated in duplicate by electrophoresis through (a) 12.5% or (b) 10% SDS-PAGE gels. After electrophoresisgels were stained with colloidal coomassie blue and each lane was cut into groups according to size of separated proteins as indicated. Each slice of gel was cut into 1 mm^3 cubes and these gel plugs were subjected to tryptic digestion before MS/MS analysis.

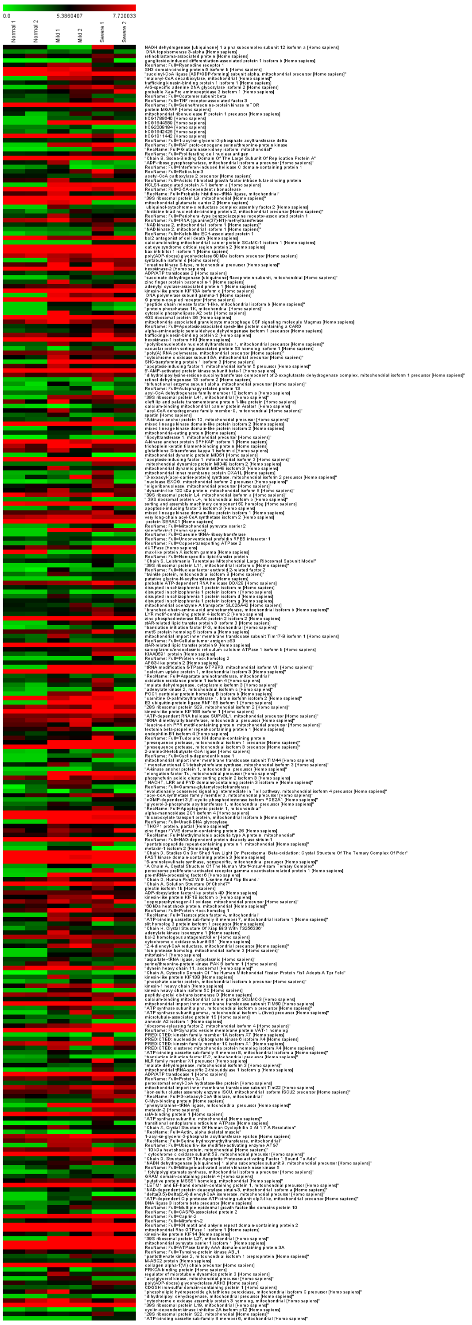

Fig C. Hierarchical clustering analysis of 288 mitochondial proteins.

Proteins were identified after GeLC-MS/MS spectra were searched against the Mitoproteome database. The data was generated by duplicate analysis of a pool of samples from 5 individuals (normal controls, mild and severe β-thalassemia/Hb E). The color scale is shown by the bar at the top.

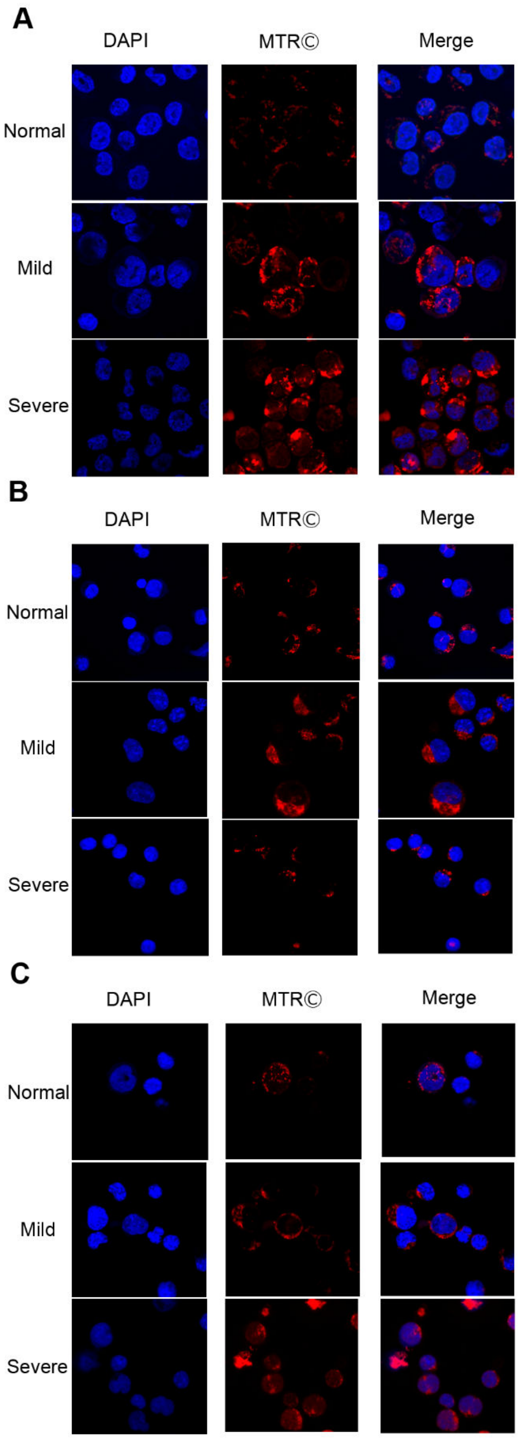

Fig D. Original unmerged and merged images from Fig 3.

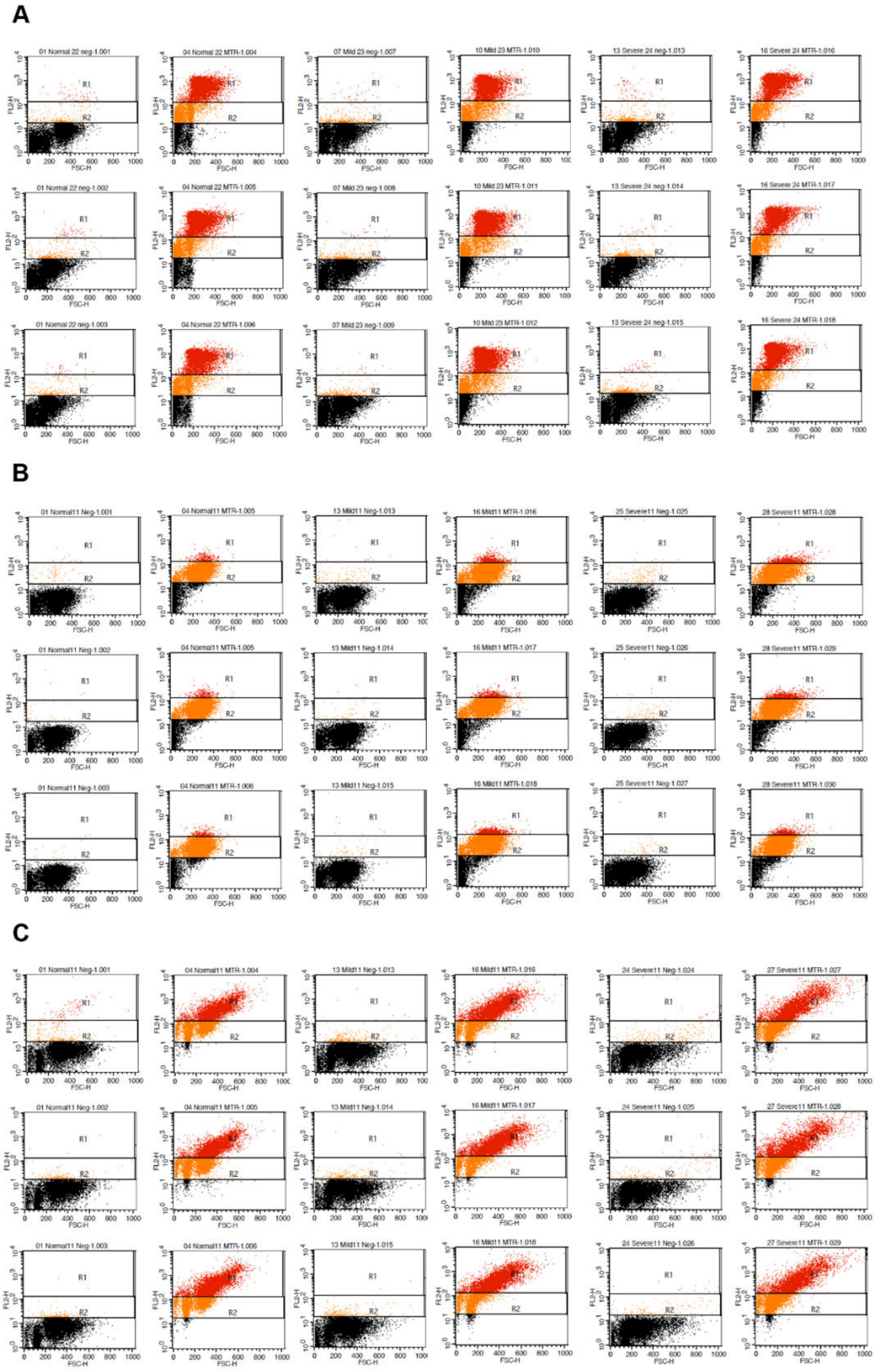

Fig E. Original scatterplots of flow cytometry as presented in Figure 3.

Table A. Specific primer sequences and cycle conditions

Gene	Primer sequence (forward and reverse)	PCR condition		Size (bp)
**mtATP6-RNA**	5'-CTCTATTGATCCCCACCTCC-3'<br>5'-TGGTAAGAAGTGGGCTAGGG-3'	95.0°C, 10 sec<br>60.0°C, 30 sec<br>72.0°C, 30 sec	40 cycles	308
**mtATP8-RNA**	5'-ATGCCCCAACTAAATACTACCG-3'<br>5'-TGGGGGCAATGAATGAAGCG-3'	95.0°C, 10 sec<br>60.0°C, 30 sec<br>72.0°C, 30 sec	40 cycles	198
**mtCYTB-RNA**	5'-GCCCTCGGCTTACTTCTCTT-3'<br>5'-AGTGATTGGCTTAGTGGGCG-3'	95.0°C, 10 sec<br>60.0°C, 30 sec<br>72.0°C, 30 sec	40 cycles	287
**Actin-RNA**	5'-ACCAACTGGGACGACATGGAGAAA-3'<br>5'-TAGCACAGCCTGGATAGCAACGTA-3'	95.0°C, 10 sec<br>60.0°C, 30 sec<br>72.0°C, 30 sec	40 cycles	192
**mtCYTB-DNA**	5'-AACTTCGGCTCACTCCTTGG-3'<br>5'-CCAATGTATGGGATGGCGGA-3'	95.0°C, 10 sec<br>60.0°C, 30 sec<br>72.0°C, 30 sec	40 cycles	377
**FRN1A-DNA**	5'-CAAACCGCTTCCATAAGGCTTTGC-3'<br>5'-CAGCGATAGCAGCCGCAGAA-3'	95.0°C, 10 sec<br>60.0°C, 30 sec<br>72.0°C, 30 sec	40 cycles	234

Table B. Significantly differentially expressed mitochondrial enrich-proteins in β^0-thalassemia/Hb E patients in erythroblast.

No.	Protein best hit	ID detail best hit
Significantly differentially up-regulated in mild β^0-thalassemia/Hb E		
1	gi|257153303	Disrupted in schizophrenia 1 protein isoform r [Homo sapiens]
2	gi|91199540	Dihydrolipoyl dehydrogenase, mitochondrial precursor [Homo sapiens]
3	gi|33636719	Mitochondrial import inner membrane translocase subunit TIM44 [Homo sapiens]
Significantly differentially up-regulated in severe β^0-thalassemia/Hb		
1	gi|339275976	A-kinase anchor protein 1, mitochondrial precursor [Homo sapiens]
2	gi|9955963	ATP-binding cassette sub-family B member 6, mitochondrial [Homo sapiens]
3	gi|148491091	calcium-binding mitochondrial carrier protein SCaMC-1 isoform 1 [Homo sapiens]
4	gi|2554941	putative glycine-N-acyltransferase [Homo sapiens]
5	gi|296439323	Protein Hook homolog 2
Significantly differentially up-regulated in both mild and severe β^0-thalassemia/Hb E		
1	gi|25306275	39S ribosomal protein L11, mitochondrial isoform c [Homo sapiens]
2	gi|7706057	39S ribosomal protein L27, mitochondrial [Homo sapiens]
3	gi|21265093	39S ribosomal protein L41, mitochondrial [Homo sapiens]
4	gi|115298648	A/G-specific adenine DNA glycosylase isoform 2 [Homo sapiens]
5	gi|8922701	Acylglycerol kinase, mitochondrial precursor [Homo sapiens]
6	gi|50345988	ATP synthase subunit gamma, mitochondrial isoform L (liver) precursor [Homo sapiens]
7	gi|7242140	ATP-dependent Clp protease ATP-binding subunit clpX-like, mitochondrial precursor [Homo sapiens]
8	gi|20140441	Autophagy-related protein 13
9	gi|334302921	Cyclin-dependent kinase 1
10	gi|167860120	Cytosolic phospholipase A2 beta [Homo sapiens]
11	gi|70995211	delta(3,5)-Delta(2,4)-dienoyl-CoA isomerase, mitochondrial precursor [Homo sapiens]
12	gi|187171277	DNA polymerase subunit gamma-1 [Homo sapiens]
13	gi|31542783	E3 ubiquitin-protein ligase RNF185 isoform 1 [Homo sapiens]
14	gi|66932984	folylpolyglutamate synthase, mitochondrial isoform a precursor [Homo sapiens]
15	gi|119592142	hCG1644589 [Homo sapiens]
16	gi|119580790	hCG1789640 [Homo sapiens]
17	gi|13435356	HCLS1-associated protein X-1 isoform a [Homo sapiens]
18	gi|530371623	kinesin family member 1A isoform X7 [Homo sapiens]
19	gi|451327636	lon protease homolog, mitochondrial isoform 3 [Homo sapiens]
20	gi|110349750	malonyl-CoA decarboxylase, mitochondrial [Homo sapiens]
21	gi|38569477	metaxin-1 isoform 2 [Homo sapiens]
22	gi|22129774	mitochondrial dynamic protein MID51 [Homo sapiens]
23	gi|45269137	mitofusin-1 [Homo sapiens]
24	gi|215820622	mixed lineage kinase domain-like protein isoform 2 [Homo sapiens]
25	gi|166795305	peptide chain release factor 1-like, mitochondrial isoform b [Homo sapiens]
26	gi|5729820	phenylalanine--tRNA ligase, mitochondrial precursor [Homo sapiens]
27	gi|315138995	POC1 centriolar protein homolog B isoform b [Homo sapiens]
28	gi|40807485	pre-mRNA-processing factor 6 [Homo sapiens]
29	gi|88758603	PRKCA-binding protein [Homo sapiens]
30	gi|167466276	protein phosphatase 1K, mitochondrial [Homo sapiens]

31	gi|239938842	Queuine tRNA-ribosyltransferase
32	gi|125651	RAF proto-oncogene serine/threonine-protein kinase
33	gi|194239664	SHC-transforming protein 1 isoform 3 [Homo sapiens]
34	gi|23618867	sideroflexin-1 [Homo sapiens]
35	gi|260763906	stAR-related lipid transfer protein 3 isoform 3 [Homo sapiens]
No.	**Protein best hit**	ID detail best hit (cont.)
36	gi|156416003	Succinate dehydrogenase [ubiquinone] flavoprotein subunit, mitochondrial [Homo sapiens]
37	gi|153090209	syntabulin isoform d [Homo sapiens]
38	gi|417324	Transcription factor A, mitochondrial
39	gi|306518614	tRNA modification GTPase GTPBP3, mitochondrial isoform VII [Homo sapiens]
40	gi|14150128	Ubiquinol-cytochrome-c reductase complex assembly factor 2 [Homo sapiens]
41	gi|62286592	Ubiquitin-like modifier-activating enzyme ATG7
42	gi|37999897	Uracil-DNA glycosylase
